# The perspectival shift: how experiments on unconscious processing don't justify the claims made for them

**DOI:** 10.3389/fpsyg.2014.01067

**Published:** 2014-09-19

**Authors:** Tom Stafford

**Affiliations:** Department of Psychology, University of SheffieldSheffield, UK

**Keywords:** unconscious processing, social priming, reasoning

## Strong claims about unconscious processing are unjustified

Recently, there has been widespread focus on studies of unconscious processing that have come out of the field of “social priming” (Doyen et al., 2012; Yong, 2012; Shanks et al., 2013). This focus has primarily been on their replicability (Pashler and Wagenmakers, 2012) and attendant claims of statistical and methodological impropriety (Simmons et al., 2011; Newell and Shanks, 2014). The logic of the claims made has received less attention. In this commentary I draw attention to certain limitations on the inferences which can be drawn about participant's awareness from the experimental methods which are routine in social priming research. Specifically, I ague that (1) a widely employed definition of unconscious processing, promoted by John Bargh is incoherent (2) many experiments involve a perspectival sleight of hand taking factors identified from comparison of average group performance and inappropriately ascribing them to the reasoning of individual participants.

The claims made for the role of unconscious processes are strong. For example, one review states “priming studies have consistently demonstrated that the mere exposure to environmental events is sufficient to directly trigger higher mental processes, in the absence of any conscious intentions or awareness that they operate” (Huang and Bargh, 2014, p. 9). The power of unconscious influences is explicitly placed in opposition to conscious processing “… by logical necessity [priming effects have] reduced the presumed causal role of intentional, conscious processes in higher mental processes” (Bargh and Huang, 2009, p. 128). This leads one review to state “some volitional behavior does not require any conscious awareness at all” (Dijksterhuis and Aarts, 2010, p. 469). Note that the claim is not that unconscious processes are involved in judgment, nor that priming can influence higher mental processes. Rather it is far stronger. Unconscious processes *produce* judgment, priming *triggers* higher mental processes, *no* conscious awareness is required.

I do not wish to question the reality of these priming effects, in that I believe that most of these studies could be replicated. Nor do I deny the challenge they pose to our folk psychology of what influences human behavior (which is often dominated by a simplistic “all acts have deliberate reasons” model). My purpose is merely to draw attention to a disjuncture between the methods used to assess unconscious processes, and the claims made for them in terms of their role in producing action.

## Problems with defining unconscious by failure to report

John Bargh has influentially defined unconscious processes as those that “do not influence subjective experience in a way that [he or she] can directly detect, understand, or report the occurrence or nature of these events” (Bargh, 1992; Bargh and Morsella, 2008; Huang and Bargh, 2014, p. 14). This definition contains a crucial ambiguity. How general must the inability to detect, understand or report be for a process to count as unconscious? Some processes, which we might most appropriately call nonconscious are forever off limits to our introspection (they are “cognitively encapsulated,” Fodor, 1983). Others may not be detected, understood or reported on just one particular occasion. Does this make them unconscious? It seems it does according to the definition promoted by Bargh.

This new definition has been used to support a shift from defining unconscious as “without awareness of the stimuli” to “without awareness of the influence of the stimuli.” This creates two problems. The first problem is it defines the “unconscious” as much by the self-model of the participants as by that of the experimenter. For example, Custers and Aarts (2005) is cited (e.g., by Huang and Bargh, 2014) as an example of subliminal priming which attests to the operation of unconscious goals. The check which was used to ensure that the stimuli really were subliminal was to ask participants at the end of the experiment if they were influenced by the stimuli (Custers and Aarts, 2005, experiment 1). In other words, unconscious operation is defined by participants denying they were influenced. Wilson (2002) has written engagingly about the divergence of our model of our thoughts and feelings from our actual thoughts and feelings. You don't need to be social psychologist to see that there could be many influences which would lead to a participant denying the influence of a stimulus on their choice, and that these might be factors which—while interesting—weaken the claim that this definition of unconscious allows us to focus on processes which are both a natural kind and truly unknown to the subjects (they may, for example, be responding to perceived social pressure to deny the influence of the factors in question).

A highly cited study (Bargh et al., 1996) reported that participants were unconsciously influenced by primes in a scrambled words task to walk more slowly down a corridor upon leaving the experiment. The authors reported, consistent with the definition of unconsciousness that I wish to question, that “no participant believes that the word has an impact on his or her behavior” (Bargh et al., 1996, experiment 1, p. 237). Remarkably, no further test of the awareness of the primes was done on the participants. Instead, a separate 19 participants were tested and funnel debriefed (with half in the experimental condition, so we can expect 9 or 10 to have experienced the elderly primes). The basis for claiming that priming was unconscious is that these participants could not predict what the influence of the primes would be, nor connect them to the elderly stereotype. Aside from issues of statistical power in this check, it seems that no participant was ever directly asked if the primes would affect the specific behavior which was measured. Even if we did ask them, we would have no strong reason to believe that the answers we got were because participants were, in some strong sense, ignorant of the influence of the primes on their behavior. Instead, they may just give answers which fit with common lay beliefs regarding which factors should and shouldn't influence behavior.

This issue of how awareness should be assessed, and of possible biases on subjective reports, is a long-standing one^1^. Reviews have highlighted the difficulty of demonstrating with certainty that a participant is unaware (Eriksen, 1960; Holender, 1986; Simons et al., 2007; Newell and Shanks, 2014). The way you operationally define consciousness is crucial to whether you can demonstrate perception without it (Reingold and Merikle, 1990; Merikle et al., 2001). In contrast to Bargh et al. (1996) other studies have used stricter methods, such as forced choice questions which remove biases to not report (since they are forced choice) and allow any feelings of awareness (however weak) to inform the choice (see Hannula et al., 2005 for a fuller discussion). It is against this background that Bargh's strategy of defining unconsciousness by failure to report should be judged (Bargh, 1992; Bargh and Morsella, 2008).

## The unjustified perspectival shift which makes claims about individual rationality based on group differences

The second problem introduced by this definition of unconscious concerns how claims of the importance of factors in individual cognition are made from experiments which compare differences in group averages. The logic of many of our behavioral experiments encourages a perspectival shift in which factors which have the major influence on each individual's choices are rendered invisible, while an experimental factor which has a minor influence on each individual's choice is highlighted. This is obviously the intent—the logic of a between subjects design is to pull out the influence of the experimental factor against a background of individual variability. Using this method we identify factors which we can show have a causal influence at the level of group average. It can be a mistake, however, to talk with confidence about the nature of an individual's choice, rather than the average effect over individuals' choices. Consider the statement “Unconscious processes have been shown to produce evaluation and social judgment” (Huang and Bargh, 2014, p. 9). This is simply wrong if we take “produce” to mean “be solely responsible for.” Unconscious processes do not produce, e.g., social judgments. The empirical foundation for this claim is experiments in which social judgment is produced by individuals, who are quite conscious of what they are doing at a macrolevel- i.e., willingly participating in an experiment. Unconscious processes are shown to influence cognitions and behaviors, but they do this as part of the conscious production of these cognitions and behaviors.

If the unconscious nature of these processes is validated at the individual level by asking participants to report what influenced their choices, but then the unconscious process itself is attested to by a difference in group means, it is possible that the experiment identifies a factor which is a minor influence on the choice as a whole. In other words the manipulation can show a strong statistical effect (and we'd hope that as professional experimenters the researchers would design a situation where this was exactly the case), but for a factor which plays a marginal role in each individual's choice. Say the experimental task is to evaluate a word as good or bad. The word is rated as good or bad and each individual, for each judgment, may decide in a way that is consonant with a deliberate and conscious decision making process (i.e., one which is completely at odds with the one being foregrounded by proponents of automatic processing). The dependent variable is reaction time, and the effect of the prime is seen in average differences in reaction time. The influence of the “unconscious” factor may be to speed or slow them in their judgment, while this judgment itself may take a value informed by reasons which the participant is fully aware of. Because “unconscious” effects are manifest this way, it is misleading to talk of the unconscious as “producing” behavior when the only thing tested are differences in characteristics of behavior. This is both because the major element of the behavior may not be affected by the experimental manipulation (e.g., in this case the judgment of the word as good or bad, rather than the speed of the judgment), and because it isn't automatic that an “unconscious” group difference implies an “unconscious” individual judgment.

This perspectival sleight of hand obscures the truly multicausal nature of behavior behind the single controlled cause that is privileged by the experimenter's perspective. Participants in these experiments are, as described, making deliberate and reasoned choices. Their failure to report the influence of the experimental factor may result from an impoverished or incorrect self-model, or it may result merely from the relative unimportance, at an individual level, of the experimental factor in guiding their choices. It is not possible, after all, to report all influences on a behavior, even for a fully informed and rational agent (the “Frame problem,” Dennett, 1978). For these reasons, it is not valid for the conclusion to be drawn that unconscious processes produce behavior, to the extent that this excludes the role of conscious processes in co-producing them. Nor is it valid to infer that unconscious processes significantly determine overall behavior of any individual at any time, as is often implied.

Evidence of *differences* due to unconscious processes at the group level do nothing to confirm the importance of the unconscious processes in affecting the *overall response* of each individual. This concern is particularly relevant for studies of unconscious processing when the criteria used to define what is unconscious are based on asking individuals to make judgments about the overall importance of factors. To explore this, consider the tension between experimental effect sizes and wider generalizability.

## Larger effect sizes can be in tension with generalisability

It is not the case that simple inspection of effect sizes will necessarily reveal the significance of an experimental factor in reasoning. Since effect sizes are based on the amount of variability in a measure, the experimenter typically selects a measure or situation in which variability in minimized. Effect sizes are maximized by situations of tight experimental control—these reduce the influence of non-experimental factors, allowing a purer measure of the experimental manipulation. Note that this means that effect sizes can be uninformative about the importance of the experimental factor in less tightly controlled situations. Indeed, there is a sense in which larger effect size (indicative of tighter experimental control) may actually anti-correlate with generalizability (which requires effects which are robust across situations). One response to failures to replications social priming studies has been that they require some expertise to set up (e.g., Bargh, 2014)—this would seem to be tacit admission of the fragile generalizability of such effects.

## Conclusion: which influences on behavior is it reasonable to expect a perfectly conscious agent to report?

The Bargh definition assumes that a rational agent with strong access to the causal mechanisms supporting their decision process could report all factors affecting their decisions. I wish to question this. It would be bizarre if individual agents had access to all the causal factors influencing each of their choices. It would be equally bizarre if they—unaware of the experimenters' interest in a particularly minor factor—were guaranteed to report it at the exact time they were asked. By shifting the defining criteria of unconscious factors to be those which are not reported we open ourselves to the risk that processes which are fully conscious, or potentially conscious, are being used to make claims about the unconscious. This may not be a problem if the revisionist definition of unconscious is born in mind at all times when the implications of these experiments are discussed. Discussion of whether or not this has been the case, both within the scientific literature and in popular discourse, is beyond the scope of this commentary.

The impoverished view of consciousness that results from the Bargh definition is supported by methods which are designed specifically to render conscious deliberation invisible. It remains to be shown that human reasoning is not dominated by self-aware deliberation and based on principles of rationality, which although limited and fallible, can be considered and improved.

### Conflict of interest statement

The author declares that the research was conducted in the absence of any commercial or financial relationships that could be construed as a potential conflict of interest.

## Footnotes

^1^I thank a reviewer for encouraging an exposition on this point.
